# Voriconazole Treatment Induces a Conserved Sterol/Pleiotropic Drug Resistance Regulatory Network, including an Alternative Ergosterol Biosynthesis Pathway, in the Clinically Important FSSC Species, *Fusarium keratoplasticum*

**DOI:** 10.3390/jof8101070

**Published:** 2022-10-12

**Authors:** Jasper E. James, Jacinta Santhanam, Richard D. Cannon, Erwin Lamping

**Affiliations:** 1 Biomedical Science Programme, Faculty of Health Sciences, Universiti Kebangsaan Malaysia, Kuala Lumpur 50300, Malaysia; 2 Sir John Walsh Research Institute, Faculty of Dentistry, University of Otago, Dunedin 9016, New Zealand

**Keywords:** FSSC, *cyp51*, *erg6*, *ebp1*, SR, AtrR, ABC transporters, azole, antifungal resistance, ergosterol biosynthesis, cholesterol biosynthesis

## Abstract

*Fusarium keratoplasticum* is the *Fusarium* species most commonly associated with human infections (fusariosis). Antifungal treatment of fusariosis is often hampered by limited treatment options due to resistance towards azole antifungals. The mechanisms of antifungal resistance and sterol biosynthesis in fusaria are poorly understood. Therefore, in this study we assessed the transcriptional response of *F. keratoplasticum* when exposed to voriconazole. Our results revealed a group of dramatically upregulated ergosterol biosynthesis gene duplicates, most notably *erg6A* (912-fold), *cyp51A* (52-fold) and *ebp1* (20-fold), which are likely part of an alternative ergosterol biosynthesis salvage pathway. The presence of human cholesterol biosynthesis gene homologs in *F. keratoplasticum* (*ebp1*, *dhcr7* and *dhcr24_1*, *dhcr24_2* and *dhcr24_3*) suggests that additional sterol biosynthesis pathways may be induced in fusaria under other growth conditions or during host invasion. Voriconazole also induced the expression of a number of ABC efflux pumps. Further investigations suggested that the highly conserved master regulator of ergosterol biosynthesis, FkSR, and the pleiotropic drug resistance network that induces zinc-cluster transcription factor FkAtrR coordinate the response of FSSC species to azole antifungal exposure. In-depth genome mining also helped clarify the ergosterol biosynthesis pathways of moulds and provided a better understanding of antifungal drug resistance mechanisms in fusaria.

## 1. Introduction

*Fusarium keratoplasticum* belongs to the *Fusarium solani* species complex (FSSC), a genetically diverse mould genus within the fungal order Hypocreales and Sordariomycetes class. Fusaria can be found in various ecological niches such as soil, water, plants, and man-made habitats. Fusaria are plant pathogens that cause significant diseases in a variety of agriculturally important crops [1,2,3]. Fusaria also frequently cause invasive mould infections (IMIs) in humans [4], and invasive fusariosis (IF) has a high mortality rate (56%) [5], especially in neutropenic patients (up to 100%) [6]. Infections caused by FSSC are problematic as most are innately resistant to commonly prescribed azole antifungals such as voriconazole (VRC) [7,8] which is one of the recommended treatment options for IF in addition to liposomal amphotericin B formulations or a combination of both [9,10,11]. Excellent guidelines for the diagnosis and management of rare mould infections, including IF, have recently been provided by the One World-One Guideline initiative [11]. Most FSSC species are susceptible to amphotericin B [7,8], but the use of this antifungal can cause side effects. Thus, there are few treatment options for immunocompromised patients with IF [12,13,14,15]. The widespread application of azole fungicides in agriculture [16] has resulted in the spread of azole-resistant *Aspergillus fumigatus* isolates [17] from the environment into hospital settings [18,19,20]. Some of these isolates exhibit resistance to multiple antifungals [21] mainly associated with *cyp51A* TR34/L98H or TR46/Y121F/T289A mutations [22,23] and, rather concerningly, they are now spreading globally [24]. A similar trend of reduced azole susceptibility in isolates of the *Fusarium solani* [25] and *Fusarium fujikuroi* [26] species complexes of agriculture and human origin has been recently reported.

Sterols play important roles in many eukaryotes including the maintenance of membrane fluidity, the regulation and distribution of membrane proteins, and the control of the cell cycle [27]. Ergosterol is the major sterol of fungal cell membranes, while the counterpart in mammalian cell membranes is cholesterol. In fungi, ergosterol depletion caused by inhibition of ergosterol biosynthesis with azole antifungals results in the accumulation of toxic sterol intermediates, disruption of cell membrane integrity and cell death [28]. Therefore, the regulation of sterol biosynthesis is essential to maintain various cellular processes and for cell survival. There are three types of transcription factors known to regulate ergosterol biosynthesis in fungi: (i) the sterol regulatory element binding proteins (SREBPs) [29] that regulate ergosterol biosynthesis in *Agaricomycotina*, *Taphrinomcyotina* and the *Eurotiomycetes*, orthologs of which also regulate cholesterol biosynthesis in *Metazoa*, including humans [30]; (ii) the sterol uptake control (Upc2) Zn_2_Cys_6_ cluster transcription factor orthologs that regulate ergosterol biosynthesis in *Saccharomycotina* yeasts [31,32]; and (iii) the sterol regulator (SR) Zn_2_Cys_6_ cluster transcription factor orthologs recently discovered in *Fusarium graminearum* and related moulds of the *Sordariomycetes* and *Leotiomycetes* lineages [33]. Interestingly, orthologs of the Zn_2_Cys_6_ cluster transcription factor named ABC transporter regulator (AtrR) were found to be key determinants of azole resistance in both the *Eurotiomycetes* species *A. fumigatus* [34,35] as well as the *Sordariomycetes* species *F. graminearum* [36]. This is somewhat surprising because ergosterol biosynthesis is regulated by SREBP and SR orthologs in *A. fumigatus* and *F. graminearum*, respectively. In fungi, all these transcription factors have been implicated in virulence and antifungal resistance.

Many antifungals target the ergosterol biosynthetic pathway. They include the azole class of antifungals that inhibit sterol 14α-demethylase encoded by *cyp51*, also known as *ERG11* in many yeast species [27,37]. Yeast species usually have only one essential *ERG11* gene, whereas moulds can have up to three *cyp51* paralogs: *Aspergillus fumigatus*, *A. niger* and *A. terreus*, for example, have two (*cyp51A*, *cyp51B*) [37] and most *Fusarium* species have three *cyp51* paralogs (*cyp51A*, *cyp51B*, *cyp51C*) [25,38]. An exception is *Aspergillus flavus* which also has three *cyp51* paralogs including *cyp51C* [39]. The Cyp51C ortholog in *Fusarium* is required for invasion of plant tissue, but was suggested to have no sterol 14α-demethylase activity [38]. The yeast ortholog Erg11 (lanosterol-14α-demethylase) catalyses the C14-demethylation of lanosterol [27] while the two Cyp51 paralogs of moulds, *Cyp51A* and *Cyp51B*, are reported to use eburicol as their preferred substrate in *A. fumigatus* [40] and *Mycosphaerella graminicola* [41]. Alcazar-Fuoli and co-authors have reported that in *A. fumigatus*, lanosterol was first converted to eburicol by C24-methyltransferase, encoded by *erg6*, and then demethylated by Cyp51, which suggests that Cyp51 should instead be named eburicol-14α-demethylase [42,43]. It is evident that such misnomers often occur for mould genes because of their homology to genes in *Saccharomyces cerevisiae*, even though the ergosterol biosynthesis pathways in the two fungi differ [44].

As *A. fumigatus* is the most common cause of IMIs, it is to date the best studied mould in terms of antifungal resistance mechanisms. It has been shown that azole resistance involves modifications and/or overexpression of the azole drug target eburicol-14α-demethylase [37] and overexpression of ATP-binding cassette (ABC) drug efflux transporters [34,35,45]. In fusaria, however, azole resistance mechanisms are still poorly understood. We recently reported a close association between a deletion in the *cyp51A* promoter and VRC resistance in both clinical and environmental FSSC isolates from Malaysia. The association between the *cyp51A* promoter deletion and VRC resistance was independent of whether they were clinical or environmental isolates. Most intriguingly, however, this association held true across species boundaries with all VRC-resistant FSSC isolates (i.e., 3 *F. keratoplasticum* and 6 *F. suttonianum* isolates) possessing the 23 bp *cyp51A* promoter deletion [25]. We also recently reported a 372-fold induction of the mRNA levels of the pleiotropic drug resistance (PDR) transporter *abc1* in response to VRC exposure [46]. When *abc1* was expressed in *S. cerevisiae* it conferred up to 1024-fold increased resistance towards a range of xenobiotics including azoles [46].

The present study describes the transcriptional response of the clinically relevant FSSC species *F. keratoplasticum* to VRC exposure. The upregulation of a group of duplicated ergosterol biosynthesis genes and the annotation of all possible sterol biosynthesis homologs/orthologs in the sequenced genomes of related FSSC species enabled the proposal of an alternative ergosterol biosynthesis salvage pathway(s) that involve human cholesterol biosynthesis orthologs. Key players of the pleiotropic drug resistance network involved in azole antifungal drug resistance that seem conserved throughout the entire FSSC were also upregulated. The phylogenetic relationship of the manually curated ergosterol biosynthesis genes of the FSSC to their *A. fumigatus* and *F. graminearum* counterparts revealed possible alternative ergosterol biosynthesis pathways for these two model species as well.

## 2. Materials and Methods

### 2.1. Fungal Isolate and Culture Conditions

*F. keratoplasticum* strain 2781 is a clinical isolate obtained from a human nail as part of routine diagnostic procedures at the Hospital Canselor Tuanku Muhriz [25]. *F. keratoplasticum* 2781 showed high MICs to itraconazole (ITC; MIC > 32 mg/L) and VRC (MIC > 32 mg/L) [25]. Although antifungal clinical breakpoints have not yet been established for fusaria, epidemiological cut-off values have been defined using the CLSI method [7]. We have previously shown that exposure to high but sub-growth inhibitory concentrations of VRC (16 mg/L) caused increased expression of *cyp51A* [25] and the PDR transporter *abc1* [46]. *F. keratoplasticum* strain 2781 was initially grown on potato dextrose agar, PDA (Merck & Co., Kenilworth, NJ, USA) at 28 °C for a week. The preparation of conidia suspensions, the growth and VRC induction, cell harvest and total RNA extraction were conducted as previously described [25]. In short, 50 mL potato dextrose broth, PDB (Merck & Co., Kenilworth, NJ, USA) was inoculated with 10 µL of *F. keratoplasticum* conidia suspension (∼1 × 10^8^ cfu/mL) and cells were grown to mid-logarithmic phase for 21 h [47]. Then, cells were incubated for an additional 80 min in the presence or absence of VRC (16 mg/L) followed by cell harvest. VRC was added to the cell suspensions as a 100× stock (1.6 mg/mL) dissolved in DMSO and the control cells were incubated with an equal volume of DMSO only. The experiment included three biological replicates.

### 2.2. Total RNA Extraction and Sequencing

Total RNA extraction (hot phenol method), DNase treatment (PureLink DNase kit; Invitrogen Inc., Carlsbad, CA, USA) and quality checks of RNA samples by agarose gel electrophoresis were conducted as previously described [25]. Total RNA samples (3 each of VRC treated and untreated control samples) were submitted to Genewiz Biotechnology Co. Ltd. (now Azenta Life Science), Suzhou, China, for transcriptome sequencing. Sample quality control checks were performed using an Agilent 2100 Bioanalyzer (Agilent Technologies, Palo Alto, CA, USA) and a NanoDrop spectrophotometer (Thermo Fisher Scientific Inc. Waltham, MA, USA). RNA sequencing (RNA-seq) of 150 bp paired-end reads with a total of ~40 million reads was performed on NovaSeq 6000 (Illumina, San Diego, CA, USA).

### 2.3. De Novo Transcriptome Assembly and Analysis of Differentially Expressed Genes (DEGs)

Quality assessment of the sequencing data was evaluated using FastQC version 0.11.4 (Simon Andrew, Cambridge, UK, https://www.bioinformatics.babraham.ac.uk/projects/fastqc/) [48] followed by the removal of adapter sequences using Cutadapt version 1.9.1 (Martin Marcel, https://cutadapt.readthedocs.io/en/v1.9.1/) [49]. The 5′ and 3′ end bases containing Ns or sequences with quality values < 20 were removed. Sequencing reads that were less than 75 bp long after trimming were also removed. De novo transcriptome assembly of the clean data was performed using Trinity version 2.2.0 (Brian J. Haas, https://github.com/trinityrnaseq/trinityrnaseq/releases) [50] followed by clustering into long non-redundant unique sequences. The prediction of coding regions within the assembled transcripts was performed using the TransDecoder version 3.0.0 (Brian J. Haas, https://github.com/TransDecoder/TransDecoder/releases?page=2) [51]. To determine their putative functions, transcripts were aligned with sequences in the following publicly available databases: NCBI non-redundant protein sequences (Nr), a manually annotated and reviewed protein sequence database (Swiss-Prot), Kyoto Encyclopedia of Genes and Genomes (KEGG), and Cluster of Orthologous Groups of proteins (COG).

To generate read densities for gene expression analysis, sequencing reads were aligned to the assembled transcripts using Bowtie2 version 2.2.6 (Langmead et al., https://sourceforge.net/projects/bowtie-bio/files/) [52] with default settings. The level of gene expression normalized to read density was calculated using RNA-seq by Expectation Maximization (RSEM) version 1.2.4 (Li et al., https://github.com/deweylab/RSEM/releases) [53] and quantified as fragments per kilobase of transcripts per million mapped reads (FPKM). The analysis of DEGs between the two groups of RNA samples was performed using the Bioconductor package DESeq2 version 1.6.3 (Love et al., https://bioconductor.org/packages/release/bioc/html/DESeq2.html) [54]. The results were further analysed to determine significant DEGs based on the criteria of fold change > 2 and a *q*-value (false discovery rate adjusted *p*-value) < 0.05.

In order to classify the biological functions of significant DEGs, GO functional enrichment analysis was performed using GOSeq [55]. Gene length and read count biases were included in the GO analysis using a filtering threshold for overrepresented sequences by applying a *p*-value ≤ 0.05.

We further compared the log2 fold-change values of *F. keratoplasticum* 2781 in response to VRC to *F. graminearum* PH-1 strains (PH-1 is a well-studied strain for azole resistance and transcriptional regulation) deleted in the transcription factor gene ΔFgSR [33] or ΔFgAtrR [36]. These data were obtained from Supplementary Data 3 (∆FgSR) in [33] and Supplementary Table S2 (∆FgAtrR) in [36], respectively. We used these log2 fold-change values to plot column clustered heatmaps by using the gplots package version 3.1.3 [56] on R version 4.2.0 (R Core Team, Vienna, Austria, https://cran.r-project.org/bin/windows/base/) [57].

### 2.4. Search for Homologs of F. keratoplasticum Genes in Other Fungi

We identified and manually curated all possible ergosterol and cholesterol biosynthesis homologs of *A. fumigatus* and *F. graminearum* and the three FSSC species *F. vanettenii*, *F. solani* FSSC5 and *F. keratoplasticum* 2781 by searching their entire genome or, in the case of *F. keratoplasticum* 2781, the entire transcriptome of cells grown either in the presence or absence of VRC using all known ergosterol or cholesterol biosynthesis protein sequences of *A. fumigatus* and *S. cerevisiae* or *H. sapiens* as query sequences (see Supplementary File S1).

Coding sequences (CDSs) within the *F. keratoplasticum* mRNA transcripts were used as query sequences with the online NCBI BLASTx to search the non-redundant protein sequence database (Nr) of *Fusarium* taxa (taxid: 5506). CDSs conserved across the *Fusarium* taxa were identified from the alignments and translated into protein sequences. The BLAST+ software version 2.13.0 [58] was used to conduct a local BLASTp search against the protein sequences of *Fusarium solani* FSSC 5 [59], *F. vanettenii* 77-13-4 [60], *F. graminearum* PH-1 [61], *A. fumigatus* Af293 [62] and *S. cerevisiae* S288C [63] downloaded from the publicly available MycoCosm [64] database (accessed on 29 June 2022) of the Joint Genome Institute (JGI). *F. solani* FSSC 5 and *F. vanettenii* 77-13-4 were selected because they are the closest relatives to *F. keratoplasticum* within the FSSC clade. The *F. keratoplasticum* genome has yet to be sequenced. *F. graminearum* is a more distant relative belonging to a neighbour clade *Fusarium sambunicum* species complex. *A. fumigatus* was included as a pathogenic mould for which azole resistance mechanisms have been well studied. *S. cerevisiae* was included as a well characterised yeast species.

### 2.5. Validation of RNA-Seq DEGs Using qPCR

First strand cDNA synthesis of 1 µg total RNA and the quantification of mRNA expression levels of individual genes was conducted by real-time qPCR as previously described [25,46]. The three *cyp51* paralogs (*cyp51A*, *cyp51B* and *cyp51C*) and *abc1* were selected for validation of the RNA-seq results [25,46]. The *erg6A* and *erg6B* paralogs encoding sterol C24-methyltransferases were also included. qPCR of three biological replicates with three technical replicates each was performed to confirm the RNA-seq results.

## 3. Results

### 3.1. De Novo Transcriptome Assembly and Annotation of Transcripts

**Figure 1:**
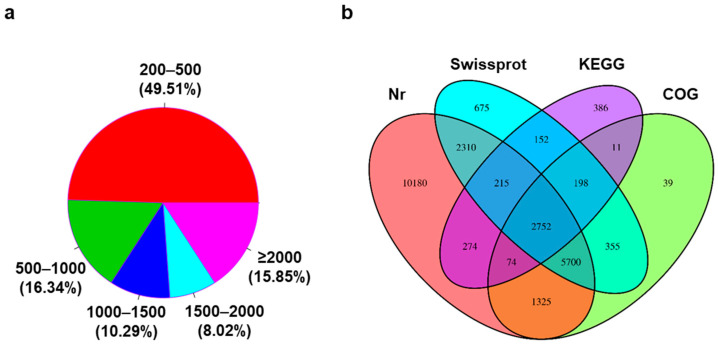
Transcript analysis. (**a**) Pie chart showing the size distribution (length in nucleotides) of the 37,274 *F. keratoplasticum* transcripts. (**b**) Venn diagram of the number of unique transcripts found in the Nr, Swissprot, KEGG and COG, databases (Nr = 22,830; Swissprot = 12,357; KEGG = 4062; COG = 10,454).

De novo transcriptome assembly of sequencing reads obtained from all six RNA samples generated a total of 9,011,979 contigs, of which 37,274 were identified as unique transcripts. The sequence length distribution of the 37,274 transcripts (Figure 1a) revealed that the majority (49.51%) were 200–500 nucleotides in length. Out of the 37,274 transcripts, 2752 had matches in all four databases Nr, Swiss-prot, KEGG and COG (Figure 1b). The Nr database had the most matches to the transcripts (22,830), and 10,180 of these transcripts were not present in any of the other three databases (Figure 1b). A similar number of transcripts were found in the Swissprot (12,357) and COG (10,454) databases, while KEGG only had matches to 4062 unique transcripts.

### 3.2. Genes Differentially Expressed in the Presence of VRC

**Figure 2:**
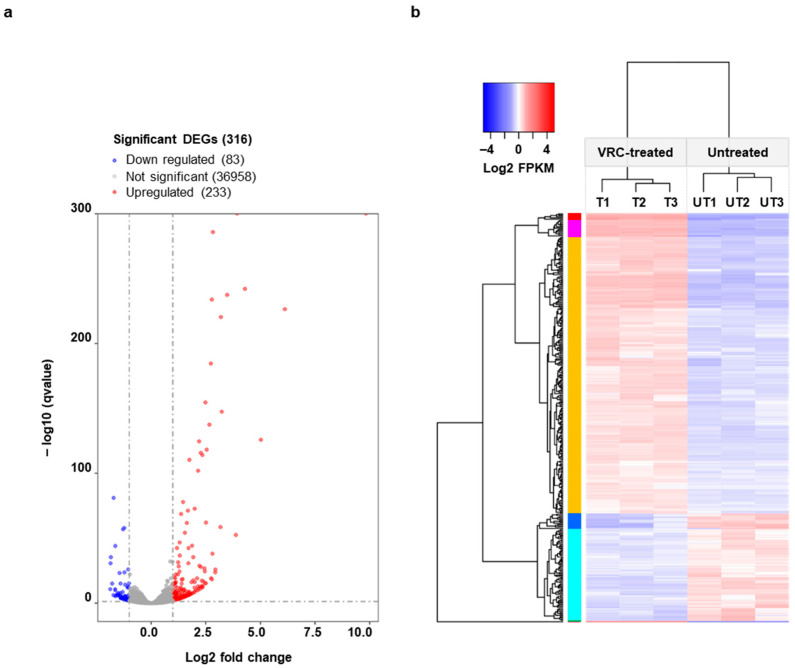
Genes differentially expressed in the presence of VRC. (**a**) Volcano plot of differentially expressed genes (DEGs) showing the log10 *q*-value (y-axis) vs. log2 fold change (x-axis). The cut-off values (dotted grey lines) used to define significant DEGs were a *q*-value of < 0.05 and a log2 fold change > |1|, i.e., larger than 2-fold. (**b**) Heatmap dendrogram (blue = down-regulation, red = up-regulation) for the normalized mRNA expression levels (log2 of FPKMs) of the 316 DEGs relative to their combined average expression levels for each of the three biological replicates grown in the absence (UT1, UT2, UT3) or presence (80 min; T1, T2, T3) of sub-growth inhibitory concentrations of VRC (16 mg/L). The hierarchical clustering indicates biologically meaningful results. Colours of the vertical bar next to the y-axis dendrogram indicate different levels of down-regulation (cyan-blue) or up-regulation (orange-magenta-red) of groups of transcripts.

A total of 316 genes were significantly differently expressed (fold-change > 2 and *q*-value < 0.05) in the presence of VRC; 233 were upregulated and 83 were downregulated (Figure 2a). To assess the reproducibility of the 316 significant DEGs in each sample from the two treatment groups (VRC-treated and untreated), we compared their FPKM profiles. Figure 2b shows that the triplicate repeats for cells grown either in the presence (T1, T2, T3) or absence (UT1, UT2, UT3) of VRC clearly grouped into two distinct clusters, confirming good reproducibility and the reliability of the results produced by the biological replicates.

**Figure 3:**
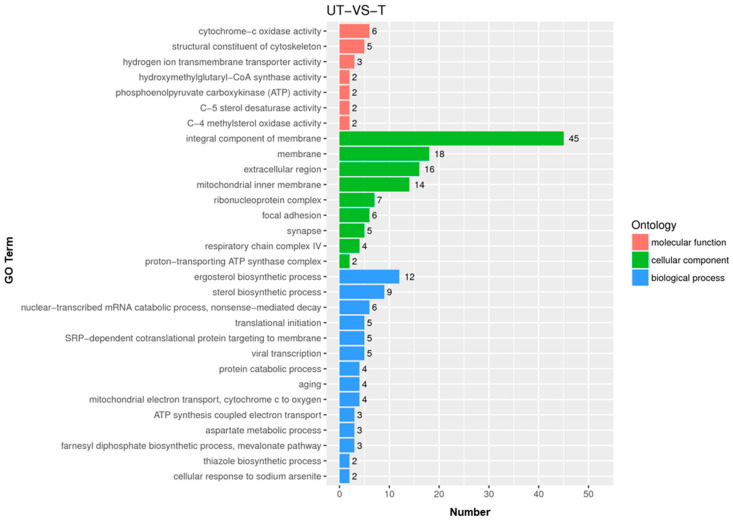
Gene ontology (GO) annotations of the 30 most prominent GO terms enriched in DEGs grouped into three main GO categories: cellular component (117), biological process (67), and molecular function (22).

Of the 316 DEGs, GO enrichment analysis revealed 117 genes were associated with various cellular components, 67 were involved in biological process and 22 were involved in molecular functions as shown in Figure 3.

### 3.3. Inventory of All F. keratoplasticum Sterol Biosynthesis Gene Transcripts and Their Orthologs in F. solani, F. vanettenii, F. graminearum, A. fumigatus and S. cerevisiae

**Figure 4:**
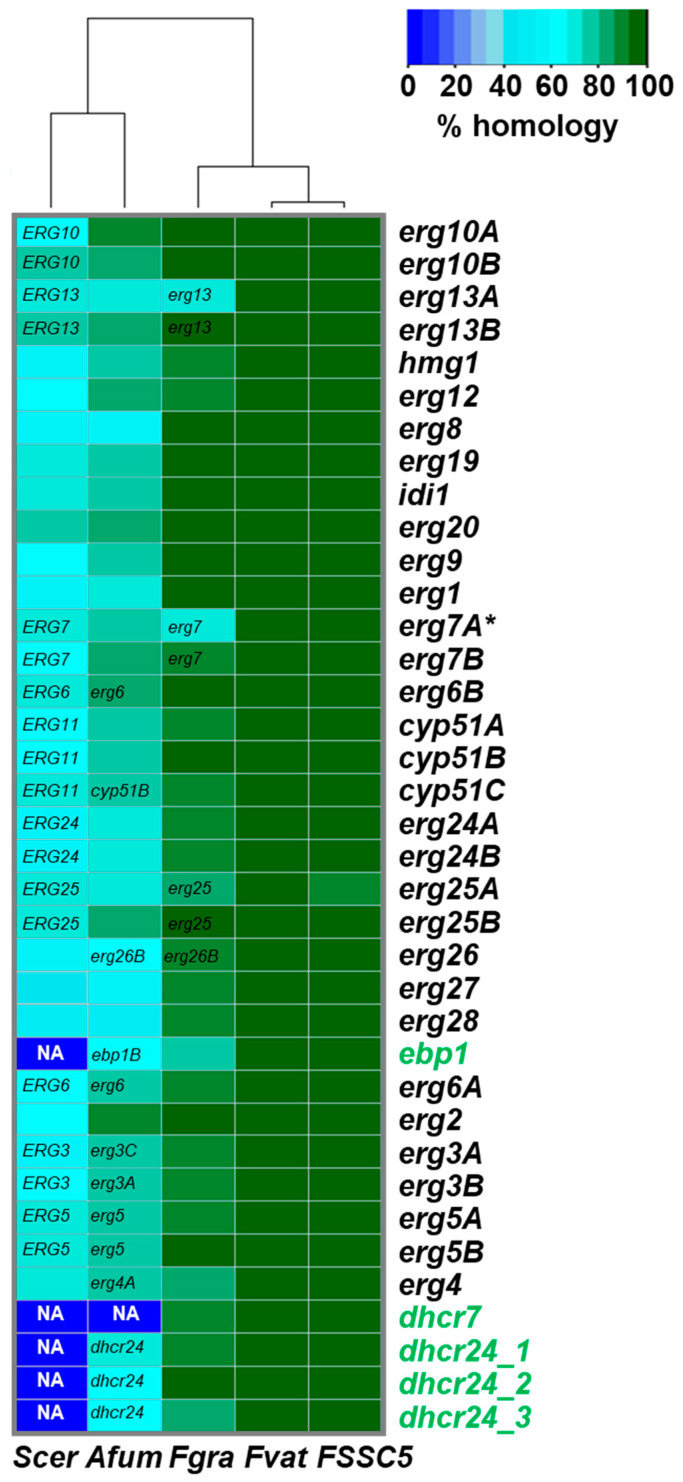
A heatmap showing the percentage homology of *F. keratoplasticum* ergosterol biosynthesis pathway protein sequences to *S. cerevisiae (Scer)*, *A. fumigatus (Afum)*, *F. graminearum (Fgra)*, *F. vanettenii (Fvat)* and *Fusarium solani* sensu stricto (FSSC5). For genes with no obvious orthologs in other fungal species the gene most homologous to the *F. keratoplasticum* gene is indicated inside the rectangle. NA indicates a homolog was not found. Genes (*ebp1*, *dhcr7* and *dhcr24*) with characteristics similar to human cholesterol biosynthesis [65] genes are highlighted in green font. Asterisk (*) indicates only a partial gene transcript was assembled/sequenced.

The search for all possible fungal ergosterol biosynthesis gene orthologs in *F. keratoplasticum* mRNA transcripts revealed the presence of eight duplicated genes (*erg10*, *erg13*, *erg7*, *erg6*, *erg24*, *erg25*, *erg3* and *erg5*) in addition to the azole drug target, *cyp51* which is known to have three paralogs in *Fusarium* [25]. In order to ‘correctly’ assign ‘A’ and ‘B’ orthologs (e.g., *cyp51A* or *cyp51B*) for the paralogs of *erg10*, *erg13*, *erg7*, *erg6*, *cyp51*, *erg24*, *erg25*, *erg3* and *erg5*, we compared *F. keratoplasticum* protein sequences with all homologous proteins from three different *Fusarium* species (*F. solani* FSSC 5, *F. vanettenii*, and *F. graminearum*), *A. fumigatus* and *S. cerevisiae* (Figure 4). The two FSSC species *F. solani* and *F. vanettenii* had the same eight duplicates as *F. keratoplasticum* and three *cyp51* genes. *A. fumigatus* had nine ergosterol biosynthesis gene duplicates (*erg10*, *erg13*, *hmg*, *erg7*, *cyp51*, *erg24*, *erg25*, *erg26* and *erg4*) and it had three *erg3* paralogs. *F. graminearum* had seven gene duplicates (*erg10*, *erg6*, *cyp51*, *erg24*, *erg26*, *erg3* and *erg5*) and *S. cerevisiae* had just one (*HMG*). After careful annotation of all ORF sequences (some introns and ATG start codons were clearly misannotated; see Supplementary File S1 for further details) their phylogenetic relationships were determined to help identify the corresponding orthologs for all six fungal species. To remain consistent with the commonly accepted nomenclature for mould *cyp51* orthologs (i.e., ‘A’ orthologs are usually expressed at low levels under normal growth conditions and are less conserved but they are upregulated in response to azole stress), ‘B’ was assigned to the more conserved ortholog across all species and ‘A’ was assigned to the less conserved gene duplicate. Thus, the *F. keratoplasticum* ‘B’ orthologs of all duplicated genes had the highest homology to their orthologs in *F. graminearum*, *A. fumigatus* and *S. cerevisiae* (Figure 4). For example, of the two *Fk-erg13* paralogs, *Fk-erg13B* had a much higher homology to *F. graminearum erg13* (95%) than did *Fk-erg13A* (69%). *A. fumigatus* also had two *erg13* paralogs, *Af-erg13A* and *Af-erg13B*. As expected, *Fk-erg13B* was significantly more conserved (81%) than *Fk-erg13A* (71%) when compared to their respective *A. fumigatus* orthologs or to *S. cerevisiae ERG13* (‘A’ 68% and ‘B’ 75%). The phylogenetic relationships between all *F. keratoplasticum* genes in the later stages of the ergosterol biosynthesis pathway, beginning with *erg7* (lanosterol synthase), and their homologs in the remaining five fungal species are presented in Supplementary Figures S1a,b. Phylogenies of the human cholesterol gene (*dhcr7* and *dhcr24)* homologs and the mevalonate pathway genes (*erg10, erg13, hmg1*) are available in Supplementary Figure S1c,d, respectively. The percentage identities and homologies for all genes across all species analysed in this study can be found in the ‘Fker FSSC2 (protein)’ sheet in Supplementary File S1.

To our surprise, we also found five genes in all three fusaria (Figure 4, green font) that were homologs of human cholesterol biosynthesis genes [65]: the ∆8,∆7-sterol isomerase homolog of emopamil binding protein (*ebp1*) [66], the 7-dehydrocholesterol reductase (*dhcr7*) and three paralogs of 24-dehydrocholesterol reductase (*dhcr24*) which suggests that fusaria can also synthesize cholesterol or cholesterol-like compounds [67]. Further investigations revealed two *ebp1* homologs and one *dhcr24* homolog, ortholog of *dchr24_1*, in *A. fumigatus* and the same five *epb1*, *dhcr7* and three *dhcr24* orthologs in *F. graminearum*. Phylogenetic trees demonstrating the evolutionary relationship between these human cholesterol biosynthesis homologs are presented in Supplementary Figure S1b,c.

### 3.4. Identification of F. keratoplasticum ABC Transporters and Transcription Factors Differentially Expressed in VRC Treated Cells

Among the 316 *F. keratoplasticum* significant DEGs, seven encoded proteins (DN33072, DN3265, DN5825, DN16847, DN36936, DN21770 and DN29261) that belonged to the ABC transporter superfamily. These proteins had homologs in other fusaria (Table 1) with percentage identities between 75–100%.

*F. keratoplasticum abc1* (DN16847) and *abc3* (DN36936) were the closest homologs of *A. fumigatus* PDR transporter *abcG1*, also known as *abcC* or *cdr1B*, a major efflux pump involved in azole resistance in *A. fumigatus* [34,35] (JGI protein ID: 1300; Supplementary File S1), with percentages identity/homology of 60/73 and 63/76 (Table 1), respectively. *F. keratoplasticum* DEGs also included transcription factors. All six (DN25544, DN12705, DN14162, DN37099, DN16099 and DN13837) differentially expressed transcription factors were also present in all other fusaria. *F. keratoplasticum atF* (DN14162) orthologs were present in *F. solani* FSSC5 and *F. vanettenii* but the *F. graminearum atF* homolog was not as highly conserved as the other five TFs, suggesting that *Fg-atF* is possibly not an ortholog of *Fk-atF* and has evolved its own unique function in *F. graminearum*. Homologs of *moc3* (DN12705), *atF* (DN14162), *SR* (DN37099) and *SREBP1* (DN13837) were not found in *A. fumigatus*. No homologs of the *F. keratoplasticum* transcription factors were found in *S. cerevisiae*.

### 3.5. Transcriptional Response of F. keratoplasticum Cells to VRC Exposure Suggests the Presence of an Alternative Route in the Ergosterol Biosynthesis Pathway

**Figure 5:**
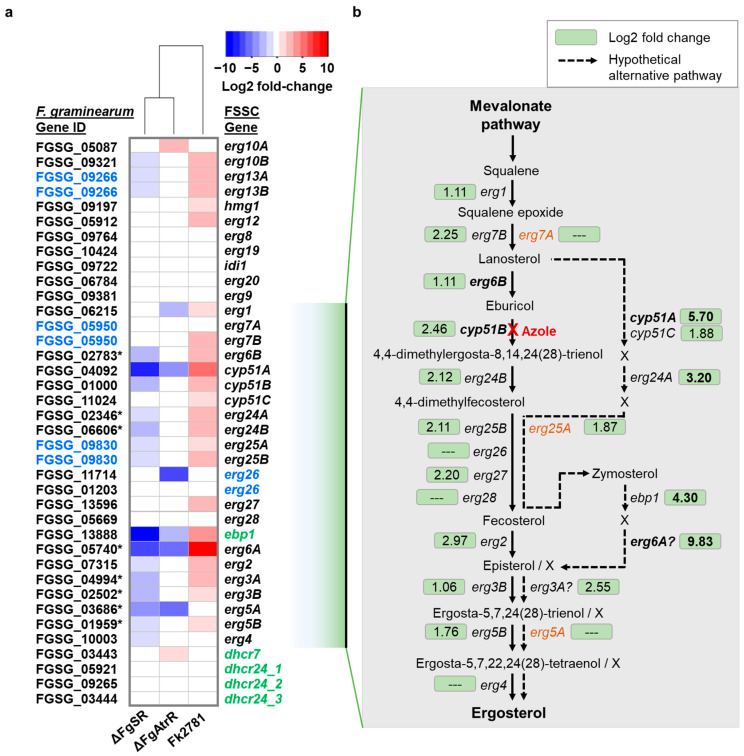
VRC exposure causes the upregulation of an alternative ergosterol biosynthesis pathway in *F. keratoplasticum*. (**a**) A heatmap showing log2 fold-change values for *F. keratoplasticum* 2781 when exposed to VRC, and effects of *F. graminearum* PH-1 *ΔFgSR* [33] and *ΔFgAtrR* [36] mutations. Genes existing as a single copy in either *F. graminearum* or FSSC are indicated in blue font. Genes that are normally involved in cholesterol biosynthesis are highlighted in green. * = *Fg-erg3*, *Fg-erg5*, *Fg-erg6* and *Fg-erg24* ‘A’ and ‘B’ paralogs were renamed to be consistent with the *F. keratoplasticum* nomenclature (see text for further details). (**b**) Proposed alternative pathway upon azole exposure is indicated by the dashed arrow line (X = to be experimentally confirmed sterol intermediates). Differential expression of genes is indicated by log2 fold-change values in the green boxes beside the genes. Dashed line (---) in green boxes indicates log2 fold change values < 1.00 and/or *p*-value > 0.05. The expression levels of *erg6A*, *erg6B*, *cyp51A*, *cyp51B*, highlighted in bold, were verified by qPCR. Genes in orange font do not take part in ergosterol biosynthesis under normal growth conditions nor in the cells’ response to azole exposure. Log2 fold change values of more than 3 are highlighted in bold numerals.

Among the 37,274 transcripts (Figure 1a), 32 were genes from the ergosterol biosynthesis pathway of which 22 were significantly upregulated (Figure 5a, red shading in the rectangles on the right column of the heatmap; and Table 2) in response to VRC. In fact, *erg6A*, *cyp51A* and, most interestingly, the human cholesterol biosynthesis ortholog, *ebp1* (Table 2), were the three most upregulated DEGs in response to VRC; 912-fold, 52-fold and 20-fold upregulated, respectively. To determine the transcription factors possibly involved in *F. keratoplasticum* 2781 ergosterol pathway gene regulation, we compared the log2 fold change values to those caused by transcription factor deletion in *F. graminearum*—strains PH-1 *ΔFgSR* [33] and *ΔFg-atrR* [36] (Figure 5a). We found that 16 out of the 22 genes in *F. keratoplasticum* upregulated in response to VRC were downregulated in *F. graminearum ΔFgSR* suggesting that FkSR is also the main transcription factor involved in the upregulation of the ergosterol biosynthesis genes in *F. keratoplasticum* upon azole antifungal treatment. The unchanged *FkSR* expression level upon VRC exposure (Table 2) is not unexpected because the *FgSR* expression levels also remained unchanged upon tebuconazole exposure of *F. graminearum* PH-1 cells [33]. The *F. keratoplasticum atrR* ortholog of *Fg-atrR*, however, was the most significantly upregulated (4.5-fold) transcription factor upon VRC exposure (Table 2). This too was expected as *Fg-atrR* transcript levels were also increased in response to tebuconazole treatment of *F. graminearum* cells [68]. The *F. graminearum ΔFg-atrR* expression profile demonstrates that FgAtrR is also involved in the upregulation of some ergosterol biosynthesis genes, but it has a smaller target range as only six genes (*erg1*, *cyp51A*, *erg26A*, *ebp1*, *erg6A* and *erg5A*) were downregulated upon its deletion (Figure 5a).

The final clue for the proposed alternative ergosterol biosynthesis pathway in Figure 5b came with the discovery of the human *ebp1* ortholog which was present in all fusaria species investigated (Supplementary File S1 and Supplementary Figure S1b). Human EBP orthologs perform the same ∆8,∆7-sterol isomerase reaction as fungal Erg2 orthologs [66]. Thus, like its human counterpart FkEbp1 most likely acts on sterol intermediates that are not methylated at their C24 position. Methylation at the C24 position, a characteristic feature differentiating ergosterol from cholesterol, is catalyzed by Erg6. In *S. cerevisiae*, Erg6 is thought to act after the Erg25/26/27/28 complex converting zymosterol to fecosterol which is the substrate for Erg2. However, mould Erg6B orthologs prefer lanosterol as a substrate [38,43] which places them before the Cyp51B orthologs in the ergosterol biosynthesis pathway (Figure 5b). These observations together with the facts that (i) *Fk-erg6A*, *Fk-cyp51A* and *Fk-ebp1* were the most dramatically upregulated genes in response to VRC; (ii) they were the only three genes, apart from *erg5A*, that were significantly downregulated upon the deletion of both *FgSR* and *Fg-atrR*; and (iii) the likelihood that *ebp1* acts on non-C24 methylated sterol intermediates led to the proposed alternative ergosterol biosynthesis pathway shown in Figure 5b. *Fk-erg7A*, *Fk-erg25A* and *Fk-erg5A* may not take part in either of these two pathways as neither is expressed under either of the growth conditions investigated (Table 2).

To compare the expression levels of each paralog in the presence and absence of VRC, we examined their FPKM values (Table 2) in each treatment group. We found that in the absence of VRC, expression levels of both *erg6A* (3) and *cyp51A* (2) were ~70 times lower than their ‘B’ paralogs (207 and 104, respectively). Conversely, when exposed to VRC, expression levels of *erg6A* (4186) and *cyp51A* (2982) were ~3 times higher than their ‘B’ paralogs (1694 and 899, respectively). This trend was not observed in the other seven (*erg10*, *erg13*, *erg7*, *erg24*, *erg25*, *erg3* and *erg5*) gene duplicates where their relative expression levels (i.e., expression level of ‘B’ paralog was higher than the ‘A’ paralog, except for *erg3* which was the opposite) were similar in both the presence and absence of VRC.

### 3.6. Differential Expression of ABC Transporters and Transcription Factors in Response to VRC

VRC induced the expression of five ABC transporters and repressed the expression of one (Table 2). Four (DN16847, DN36936, DN21770, DN29261) were PDR transporters of the ABCG subfamily and two (DN3265 and DN33072) were ABCB-type ABC transporters (Table 2). PDR transporters *abc1* (DN16847), *abc3* (DN36936), orthologs 1 and 3 of 9 *F. vanettenii* cluster B PDR transporters [46], and DN21770, ortholog 3 of 3 *F. vanettenii* cluster H1 PDR transporters [46], were 4.9-, 2.8- and 2.4-fold induced and the PDR transporter DN29261, ortholog 2 of 2 *F. vanettenii* cluster F PDR transporters [46], was 3.6-fold downregulated in response to VRC. DN16847 encodes the previously characterized *F. keratoplasticum* multidrug efflux pump, *abc1* [46], orthologs of which are involved in virulence and/or azole drug resistance in the important fungal pathogens *F. vanettenii* (also known as *Nectria haematococca*) [70], *F. graminearum* [71] and *F. culmorum* [72]. *Fk-abc3* is closely related to *Fk-abc1* [46]. The *F. keratoplasticum abc1* orthologs in *F. graminearum* (FGSG_08312; 81% protein identity) [36] and *A. fumigatus* (*abcG1*, also known as *abcC* or *cdr1B*; 60% protein identity) [34,35,45] are also involved in azole resistance. Deletion of *cdr1B* in *A. fumigatus* [73] caused increased ITC, but not VRC, susceptibility. The function of cluster F PDR transporters, ancestors of all fungal PDR transporters [69], and the possible involvement of the ABCB-type ABC transporter homologs DN3265 and DN33072 in azole resistance remains to be explored.

Five transcription factors were differentially expressed in *F. keratoplasticum* when exposed to VRC of which three (DN25544, DN12705, DN14162) were up-regulated and two (DN16099, DN13837) were down-regulated (Table 2). Interestingly, the highest (4.4-fold) upregulated transcription factor DN25544 is clearly the ortholog of *atrR*, an autoregulatory Zn_2_-Cys_6_ zinc cluster transcription factor and master regulator of *cyp51* orthologs and/or ABC transporters involved in azole resistance in *F. graminearum* [36] and *A. fumigatus* [34,35]. The transcription factor DN13837, which was downregulated 2.2-fold, is a sterol regulatory element binding protein 1 (SREBP1) homolog; these proteins are the main regulators of sterol biosynthesis in many fungal species (e.g., SrbA in *A. fumigatus* [74]) and in higher eukaryotes including humans [75]). DN16099 (2.1-fold downregulated) is an AP-1 like transcription factor (*yap5*), homologs of which are involved in stress response and yeast iron regulatory mechanisms [76].

### 3.7. Correlation between Gene Expression Levels Measured by qPCR and RNA-Seq

**Figure 6:**
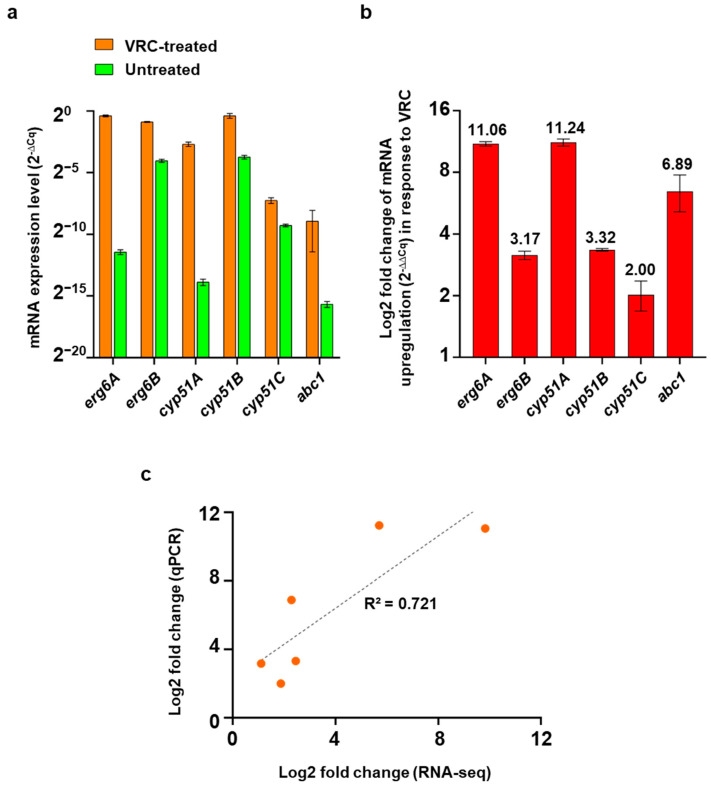
Quantification of *F. keratoplasticum* gene expression levels. (**a**) qPCR quantification of *F. keratoplasticum* *erg6A*, *erg6B*, *cyp51A*, *cyp51B*, *cyp51C* and *abc1* mRNA expression levels (2^−ΔCq^) normalised with *gpd1* and (**b**) Log2 fold increased expression (2^−ΔΔCq^) of logarithmic cells in response to 80 min exposure to VRC (16 mg/L), numbers on top of the bars indicate log2 fold changes. (**c**) Correlation between the log2 fold change values obtained using qPCR (Y-axis) and RNA-seq (X-axis).

The expression levels for key genes identified from the RNA-seq analysis were measured by qPCR. The Cq values were normalized with *GAPDH* (*gpd1*, Figure 6a), and their fold up- or down-regulation in response to VRC (Figure 6b) was determined using the 2^−ΔΔCq^ method [77]. Clear differences in expression levels (Figure 6a) were observed between the cells grown in the presence and absence of VRC. The qPCR analysis revealed a 2100-fold upregulation of *erg6A*, a 9-fold upregulation of *erg6B* and a 2400-, 10- and 4-fold upregulation of *cyp51A*, *cyp51B* and *cyp51C*, respectively, and a 120-fold upregulation of *abc1* in response to VRC (Figure 6b). These VRC responses showed similar trends to the expression levels determined by RNA-seq (R^2^ = 0.712; Figure 6c). In particular, *erg6A* and *cyp51A* were both expressed far less (i.e., 2142-fold and 2431-fold less), at almost undetectable levels, than their ‘B’ paralogs under normal growth conditions, but they were induced much more than their ‘B’ paralogs in response to VRC so that they reached comparable (Figure 6) or even 2- to 3-fold higher (Table 2) expression levels than their ‘B’ paralogs in the qPCR and RNA-seq experiments, respectively.

## 4. Discussion

A recent landmark study by Liu et al. [33] highlighted a novel transcription factor in *F. graminearum*, FgSR, which binds to a 16 bp *cis*-element present in the promoter of 119 genes, 20 of which were involved in ergosterol biosynthesis including *cyp51A*, *cyp51B*, *cyp51C*, *erg6A*, and *erg6B.* Fourteen of these 20 genes were also significantly downregulated in the ∆FgSR strain [32]. *Fg-erg5A* and *Fg-erg4* were two notable exceptions. Although FgSR did not bind to either promoter, both genes were also significantly downregulated in the ∆FgSR strain [32]. Deletion of *FgSR* in *F. graminearum* had an inverse effect to that of VRC exposure on all these orthologs in *F. keratoplasticum* (Figure 5a). Again, the only exceptions were *erg5A* and *erg4* that were either not expressed under either growth condition (*erg5A*) or fell just below (0.98; *erg4*) the log2-fold change threshold of 1.0 (Supplementary File S1). These striking similarities in the regulation of each of these ergosterol biosynthesis orthologs indicates that FkSR is also likely the key transcription factor that regulates ergosterol biosynthesis in *F. keratoplasticum*, and it too possibly functions via phosphorylation mediated by the kinases of the high osmolarity glycerol response (HOG) pathway [33]. A similar 16 bp *cis*-element binding site is present in the *cyp51A* and *cyp51B* but not the *cyp51C* promoters of *F. keratoplasticum* [25]. We also found this motif in all *cyp51A* and *cyp51B* promoters of two other FSSC species [25] as well as in *F. graminearum*, *F. verticillioides*, and *F. oxysporum* [33]. As expected, *FkSR*, the ortholog of *FgSR* (Table 2), was not differentially expressed by VRC exposure which is consistent with the findings in Liu et al. [33] where *F. graminearum* cells exposed to tebuconazole or depleted in ergosterol in a mutant deleted of *erg4* did not alter FgSR protein levels. This confirms that the novel sterol regulator SR is indeed likely to be a characteristic feature of all *Leotiomycetes* and *Sordariomycetes* species [33].

*Fk-atrR* was the transcription factor most significantly upregulated (4.4-fold; Table 2) in response to VRC. FkAtrR is clearly the ortholog (63% protein identity) of AfAtrR (ABC transporter regulator; their phylogenetic relationship is shown in Figure 1 of [36]) that mediates resistance to azoles in *A. fumigatus* by possibly autoregulating its own expression [33] and co-regulating *srbA*, *cyp51A* and *abcG1/cdr1B* expression [34,35,45]. AtrR orthologs act similarly in fusaria species. Deletion of *F. graminearum atrR* (FGSG_06810; 84% protein identity) [36] caused the downregulation of *cyp51A* (FGSG_04092; 19-fold), *erg5A* (FGSG_03686; 29-fold) and *erg6A* (FGSG_05740; 46-fold) which are clearly the orthologs of *Fk-cyp51A* (81% protein identity), *Fk-erg5A* (80%) and *Fk-erg6A* (86%), respectively (Supplementary Figure S1a,b). Unfortunately, FGSG_03686 and FGSG_05740 were named *Fg-erg5B* and *Fg-erg6B* and not *Fg-erg5A* and *Fg-erg6A*, respectively, in that publication, even though, like for the *Fg-cyp51A* and *Fg-cyp51B* orthologs, they are less well conserved than *Fg-erg5B* (FGSG_01959) and *Fg-erg6B* (FGSG_02783) and expressed at much lower levels than *Fg-erg5B* and *Fg-erg6B* under normal growth conditions and two of the most downregulated genes in the ∆FgSR strain (Figure 5a and ‘Fgra (gene)’ sheet in Supplementary File S1). Thus, for consistency and to avoid confusion, we modified the nomenclature for all *F. graminearum* ergosterol biosynthesis gene duplicates so that all fusaria orthologs are labelled with the same ‘A’ (salvage pathway) or ‘B’ (default pathway) extensions (Figure 4 and Figure 5b). *Aspergillus* species have only one *erg6* ortholog which appears to be the common ancestor of all fusaria *erg6B* orthologs (Supplementary Figure S1b). As expected for the FgAtrR ortholog of AfAtrR which possibly autoregulated its own expression and co-regulated *srbA* expression in *A. fumigatus* [34], *Fg-atrR* expression was not affected in the ∆FgSR knock-out strain [33], but it was upregulated (3.3-fold) in response to tebuconazole [68], which is very similar to our findings with *Fk-atrR* being 4.4-fold upregulated in response to VRC.

Previous studies found the *F. graminearum Fk-abc3* ortholog FGSG_08312 was down-regulated 4.5-fold in the ∆AtrR strain [36] and upregulated 6.4-fold in response to tebuconazole treatment [68]. *Fk-abc3* was the second highest upregulated (2.8-fold) transporter in this study, after *Fk-abc1* (4.9-fold). *A. fumigatus abcG1* [34,35,45] is the closest homolog of both *Fk-abc1* and *Fk-abc3* (60% and 63% protein identity, respectively; Table 1). Interestingly, *Fg-abc3* (FGSG_04580) [71], the ortholog (81% identical) of *Fk-abc1* [46], was not significantly down-regulated in *F. graminearum* strain ΔAtrR [36] and not upregulated when *F. graminearum* was treated with tebuconazole [68]. Thus, *F. keratopasticum* AtrR possibly co-regulates the expression of *abc3*, but not *abc1*, with *cyp51A* as was reported for their respective orthologs or closest homologs in *F. graminearum* [36] and *A. fumigatus* [34,35,45], respectively. *Fk-abc1* orthologs in a number of fungal plant pathogens have been implicated in azole resistance and virulence by protecting cells against azoles and phytoalexins [46,71]. Thus, both the *abc1* and *abc3* orthologs are likely to be involved in azole antifungal drug resistance in *F. keratoplasticum* and related fungal species.

To date, detailed mechanisms of ergosterol biosynthesis remain underexplored in mould pathogens like fusaria. The discovery of the human ∆8,∆7-sterol isomerase ortholog *ebp1* in *F. keratoplasticum* and all related FSSC species, and even two paralogs *ebp1A* and *ebp1B* in *A. fumigatus*, was rather surprising given that fungi have evolved their own characteristic ∆8,∆7-sterol isomerase *erg2*. Plants and animals, on the other hand, use *EBP* orthologs for the ∆8,∆7-sterol isomerase reaction [78,79]. Even though murine EBP could complement a yeast mutant deleted in *ERG2* which exhibited a very similar sterol composition to wildtype *S. cerevisiae*, murine EBP and ScErg2 share no similarities and are phylogenetically completely different proteins [66]. *erg2* encodes a typical bitopic membrane protein with an N-terminal transmembrane span while *Fk-ebp1* and all related fungal *ebp1* homologs encode proteins with five predicted transmembrane spans, respectively (Supplementary File S1). Perhaps this is why these *ebp1* homologs have been overlooked for so long in the model moulds *A. fumigatus* and *F. graminearum*.

Because *Fk-ebp1* was one of the sterol biosynthesis genes most highly upregulated in response to VRC and because its presence forced us to ascertain how it might possibly fit into the commonly assumed ergosterol biosynthesis pathway in *F. keratoplasticum*, we were interested to find out whether this enzyme had simply been missed in previous investigations, by searching their data. That was indeed the case. The *F. graminearum ebp1* ortholog FGSG_13888 was actually the most dramatically down-regulated sterol biosynthesis gene (919-fold) in the ∆FgSR strain (we found it among the list of DEGs in the Supplementary Data 3 file of [33]) and also one of the most down-regulated (8.6-fold) sterol biosynthesis genes in the ∆FgAtrR strain (we found it among the list of DEGs in the Supplementary Table S2 of [36]). The *cyp51A* and *ebp1* orthologs were also the highest upregulated genes (163-fold each) in response to exposing *F. graminearum* cells to tebuconazole [68]. In addition, one of the two *A. fumigatus* orthologs, *ebp1B*, was one of only four sterol biosynthesis genes (i.e., *erg24A*, *erg25A*, *erg3B* and *ebp1B*) that was dramatically downregulated in a ∆SrbA strain when the cells were grown under hypoxic growth conditions [74]. Despite its obvious significance, *ebp1* was not recognized as the ‘missing link’ that it appears to be for an alternative ergosterol biosynthesis salvage pathway that is induced by azole antifungal treatment of fusaria and possibly also *A. fumigatus*. However, the rather different set of *erg* paralogs in *A. fumigatus* suggests a different salvage pathway is induced by azole exposure in this *Eurotiomycetes* model species [42,80].

We propose two major changes to the ergosterol biosynthesis pathway in response to the inhibition of *cyp51B* by VRC. Erg6A acts after Ebp1 because it possibly prefers non-C24 methylated substrates, like its mammalian homologs (C24 methylation is a typical feature of fungal and plant sterols), and Ebp1 acts after the Erg25/26/27/28 complex right where it is placed in the cholesterol biosynthesis pathway. *Cyp51A* possibly prefers lanosterol as a substrate. *erg7A*, *erg25A* and *erg5A* (in orange fonts in Figure 5b) possibly play no part in either pathway because their expression levels are simply too low under either growth condition (i.e., with and without VRC). Erg24A and Erg24B are both upregulated to metabolise both types of sterol intermediates, those that are C24 methylated by Erg6B and processed by the only partially inhibited Cyp51B (*F. keratoplasticum* 2781 had MIC_VRC_ > 32 mg/L [25]) and those that are not methylated. These two intermediates are further processed by the Erg25B/26/27/28 complex which can process both types of intermediates to generate fecosterol and zymosterol, respectively. Fecosterol and zymosterol are then processed by the two ∆8,∆7-sterol isomerases Erg2 and Ebp1, respectively. Further progression in the two pathways is not clear. However, because there are two *erg3* paralogs, both of which are expressed at similar levels under normal growth conditions (Table 2) and *erg3A* is significantly more induced by VRC (5.9-fold) than *erg3B* (2.1-fold), there are possibly two separate pathways leading all the way to the production of ergosterol. The final four enzymatic reactions necessary for ergosterol biosynthesis may involve a different series of intermediate steps (hence the question marks for the placements of *erg6A* and *erg3A* and an X for different intermediates in the proposed salvage pathway in Figure 5b). Three alternative pathways were proposed for the conversion of fecosterol to ergosterol in *A. fumigatus*. Each pathway involved a different series of enzymatic reactions carried out by Erg2, three Erg3, Erg5 and two Erg4 paralogs, each preferring different intermediates as their respective substrates (see Figure 3 in [42]). In all three of these proposed pathways the first step involved the conversion of fecosterol to episterol by Erg2. However, our discovery of two *ebp1* paralogs, one of which, *ebp1B*, and *erg3B* were required for adaption to hypoxic growth [74], suggests one or both of these *ebp1* paralogs may play a part in these alternative pathways.

The possible contribution of the third Cyp51C paralog to ergosterol biosynthesis remains to be investigated even though it was claimed to have no sterol 14-α demethylase activity [38]. This assumption was based on the fact that *Fg-cyp51A* and *Fg-cyp51B*, but not *Fg-cyp51C*, could complement a *S. cerevisiae* mutant deleted in *ERG11*. However, the authors did not explore whether *Fg-cyp51C* was actually expressed nor did they consider the possibility that FgCyp51C may not accept lanosterol as its preferred substrate. The same authors also created null-mutants for all three *Fg-cyp51* paralogs and the double deletion mutants for the A,C and B,C paralog combinations. However, the authors did not create a double deletion mutant for the A,B paralog combination because they “considered it to be lethal” [38]. Yet, all indications are that *cyp51C* is part of the ergosterol biosynthesis pathway. It too is upregulated in response to azole stress, deletion of *Fg-cyp51C* also affected the sterol composition of *F. graminearum* cells and it was required for virulence on host wheat ears [38]. It is therefore conceivable that a *Fg-cyp51A*/*Fg-cyp51B* double deletion mutant is viable and possibly uses another alternative ergosterol biosynthesis pathway to produce enough ergosterol or sterol intermediates for cell survival. *F. keratoplasticum erg7A*, *erg25A* and *erg5A* were not expressed under either growth condition tested, yet they are conserved in all FSSC species investigated. This suggests the possibility of an additional ergosterol biosynthesis salvage pathway that may be induced by hypoxia or other growth conditions not tested in the current study.

Dhcr7 and Dhcr24 catalyse the final two steps of cholesterol biosynthesis by reducing the double bonds at C7 and C24, respectively [67]. The presence of one *dhcr7* and three *dhcr24* orthologs in fusaria (Figure 4) indicates that fusaria may also be able to produce cholesterol or cholesterol-like compounds under certain stress conditions. A viable ‘cholesterol yeast’ was successfully engineered by deletion of *S. cerevisiae ERG5* and *ERG6* and replacing them with fish *DHCR7* and *DHCR24*, respectively [81]. Chytridiomycota are examples of fungi that naturally synthesise cholesterol and other Δ5 sterols as their predominant membrane sterols [82]. In addition, an entire cholesterol biosynthesis pathway has also recently been reported for tomato plants that can produce significant quantities of cholesterol [79]. *Fk-dhcr7* and *Fk-dhcr24_3* were not expressed to any significant amount under either of the two growth conditions investigated. However, *Fk-dhcr24_2*, and to a lesser extent *Fk-dchr24_1*, were expressed at reasonable levels (Table 2) indicating they are functionally relevant under normal growth conditions. Perhaps *F. keratoplasticum* produces cholesterol or cholesterol-like compounds at higher temperatures or during host invasion by upregulating *Fk-dhcr7* and *Fk-dhcr24_3* and downregulating *Fk-erg5B* and *Fk-erg6B*, respectively. Cholesterol may provide a more rigid membrane environment enabling cells to grow at higher temperatures. No further possible plant, fungal or human sterol biosynthesis homologs were found in any of the fungal genomes of *F. graminearum*, *F. solani* FSSC5 or *F. vanettenii*.

## 5. Conclusions

The highlight of this study is the discovery of mould *ebp* homologs that are possibly an integral part of an alternative ergosterol biosynthesis salvage pathway that is upregulated in fusaria species upon azole antifungal exposure. VRC exposure of *F. keratoplasticum* also caused the upregulation of a number of ABC transporters including the major multidrug efflux pump genes *abc1* and *abc3*. The antifungal drug resistance response of *F. keratoplasticum* appears rather well conserved in all fusaria species with orthologs of the sterol biosynthesis master regulator, SR, and the pleiotropic drug resistance regulator, AtrR, working together to protect fusaria from the effects of azole antifungals. We also discovered the elements of a conserved cholesterol biosynthesis pathway that may be expressed at higher temperature or under different stress conditions. Future biochemical studies are required to confirm these discoveries.

## Figures and Tables

**Table 1:** Fungal orthologs or closest homologs of proteins encoded by *F. keratoplasticum* DEGs. Results of BLASTp searches with *F. keratoplasticum* gene products as queries against protein sequences of *F. solani* FSSC5, *F. vanettenii* 77-13-4, *F. graminearum* PH-1, *A. fumigatus* Af293 and *S. cerevisiae* S288C obtained from the Joint Genome Institute (JGI) MycoCosm database.

	*F. keratoplasticum 2781*	JGI Protein ID (% Identity/% Homology) ^1, 2^				
Gene		*Fusarium solani* FSSC 5	*F. vanettenii* 77-13-4	*F. graminearum* PH-1	*A. fumigatus* Af293	*S. cerevisiae* S288C
ABC transporters						
ABCB-1	DN33072	511443 (100/100)	96330 (98/99)	6063 (80/88)	3538 (54/72)	3617 (28/47)
ABCB-2	DN3265	543122 (100/100)	92751 (99/100)	7949 (75/85)	8558 (47/66)	6078 (28/48)
ABCC-*yor1*	DN5825	393338 (100/100)	63546 (98/100)	8483 (85/92)	5371 (59/74)	2864 (37/56)
ABCG-*abc1*	DN16847	537064 (100/100)	63187 (98/99)	3418 (81/89)	1300 (60/73)	1445 (46/64)
ABCG-*abc3*	DN36936	417245 (100/100)	35467 (99/100)	9970 (86/91)	1300 (63/76)	5806 (49/67)
ABCG-H1	DN21770	439905 (100/100)	35868 (99/100)	9973 (84/91)	6736 (64/78)	6299 (37/56)
ABCG-F	DN29261	57426 (99/99)	68948 (99/99)	12739 (81/90)	4105 (56/72)	5566 (38/58)
Transcription factors						
*atrR*	DN25544	393104 (100/100)	96223 (98/99)	7871 (84/90)	1901 (63/73)	NA
*moc3*	DN12705	489853 (98/99)	1518 (97/98)	8308 (66/76)	NA	NA
*atF*	DN14162	502182 (96/98)	84321 (100/100)	9383 (36/55)	NA	NA
*SR*	DN37099	384730 (100/100)	103092 (99/100)	1375 (87/93)	NA	NA
*yap5*	DN16099	42136 (99/100)	74330 (97/98)	1660 (73/82)	1198 (46/59)	NA
*SREBP1*	DN13837	554245 (100/100)	67823 (99/100)	5412 (84/91)	NA	NA

^1^ Protein ID was obtained from the Joint Genome Institute (JGI) MycoCosm database of *Fusarium solani* FSSC 5 [59], *F. vanettenii* 7713-14 [60], *F. graminearum* PH-1 [61], *A. fumigatus* Af293 [62] and *S. cerevisiae* 288C [63]. ^2^ NA indicates no homolog was found.

**Table 2:** Expression levels of DEGs involved in ergosterol biosynthesis, drug efflux and gene regulation which could contribute to *F. keratoplasticum* azole resistance. Log2 fold-change and mean FPKM values of all other significant DEGs are available in Supplementary File S2.

Gene ID	Gene Description ^1^	Gene	Log2 Fold Change ^2, 3, 4^	Mean FPKM Values	
				VRC-Treated (*n* = 3)	Untreated (*n* = 3)
Ergosterol biosynthesis ^5^					
DN33281	Acetyl CoA thiolase	*erg10A*	−0.41	99	119
DN17604	Acetyl CoA thiolase	*erg10B*	2.49	1165	180
DN37027	HMG CoA synthase	*erg13A*	2.44	209	25
DN8815	HMG CoA synthase	*erg13B*	2.78	1199	151
DN33169	HMG CoA reductase	*hmg1*	1.64	208	59
DN18060	Mevalonate kinase	*erg12*	2.28	110	20
DN33074	Phosphomevalonate kinase	*erg8*	* −0.10	30	29
DN29440	Mevalonate pyrophosphate kinase	*erg19*	* −0.03	90	83
DN29378	Isopentenyl pyrophosphate isomerase	*idi1*	* −0.16	171	172
DN10425	Geranyl pyrophosphate synthase	*erg20*	* 0.17	20	16
DN17806	Squalene synthase	*erg9*	0.88	343	166
DN18090	Squalene epoxidase	*erg1*	1.11	78	31
DN26925	Lanosterol synthase	*erg7A*	* 0.08	2	0
DN37088	Lanosterol synthase	*erg7B*	2.25	59	11
DN33059	Sterol C-24 methyltransferase	*erg6B*	1.11	1694	207
DN25638	Eburicol 14-α demethylase	*cyp51A*	5.70	2982	2
DN21545	Eburicol 14-α demethylase	*cyp51B*	2.46	899	104
DN8317	Claimed not to encode sterol 14-α demethylase ^6^	*cyp51C*	1.88	20	5
DN29591	Sterol C-14 reductase	*erg24A*	3.20	160	15
DN32963	Sterol C-14 reductase	*erg24B*	2.12	290	58
DN8081	Sterol C-4 methyl oxidase	*erg25A*	1.87	2	0
DN17697	Sterol C-4 methyl oxidase	*erg25B*	2.11	1543	248
DN29406	Sterol C-3 dehydrogenase	*erg26*	0.35	82	58
DN3112	3-keto sterol reductase	*erg27*	2.20	149	28
DN22157	Sterol C-4 demethylase	*erg28*	* 0.06	68	58
DN33250	Sterol C-8 isomerase	*ebp1*	4.30	152	6
DN32910	Sterol C-24 methyltransferase	*erg6A*	9.83	4186	3
DN25482	Sterol C-8 isomerase	*erg2*	2.97	1039	109
DN15167	Sterol C-5 desaturase	*erg3A*	2.55	364	52
DN37320	Sterol C-5 desaturase	*erg3B*	1.06	103	24
DN16841	Sterol C-22 desaturase	*erg5A*	* 0.30	0	0
DN10857	Sterol C-22 desaturase	*erg5B*	1.76	575	151
DN21765	Sterol C-24 reductase	*erg4*	0.98	170	77
DN12846	C-7 sterol reductase	*dhcr7*	NA	0	0
DN10241	C-24 sterol reductase	*dhcr24-1*	NA	9	8
DN18330	C-24 sterol reductase	*dhcr24-2*	NA	27	29
DN14443	C-24 sterol reductase	*dhcr24-3*	NA	1	1
ABC transporters ^7^					
DN3265	ABCB family	-	1.38	4	1.4
DN33072	ABCB family	-	1.25	20	5.4
DN16847	ABCG family (PDR transporter; cluster B)	*abc1*	2.29	1	0.05
DN36936	ABCG family (PDR transporter; cluster B)	*abc3*	1.48	21	3.3
DN21770	ABCG family (PDR transporter; cluster H1)	-	1.24	17	6.4
DN29261	ABCG family (PDR transporter; cluster F)	-	–1.86	3	11.9
Transcription factors					
DN25544	Zn2-Cys6 transcription factor ^8^	*atrR*	2.15	34	7
DN12705	Transcriptional regulatory protein	*moc3*	1.32	5	2
DN14162	Terreic acid cluster-specific transcription factor	*atF*	1.05	3	1
DN37099	Ergosterol biosynthesis regulator in fusaria ^9^	*SR*	0.13	33	27
DN16099	AP-1-like transcription factor	*yap5*	–1.08	0	2
DN13837	Sterol regulatory element-binding protein 1	*SREBP1*	–1.16	4	15

^1^ Gene descriptions are based on the Uniprot descriptions except for genes with citations. ^2^ Negative values are the log2 values for downregulated genes. ^3^ DEGs with log2 fold changes above 2.00 are highlighted with a grey background. ^4^ Asterisk (*) indicates no significant change in expression (log2 fold changes with a *q*-value of > 0.05). ^5^ Genes are listed according to their predicted position in the ergosterol biosynthesis pathway (see text for further details). ^6^ Fan et al. [38] claimed that *F. graminearum cyp51C* does not encode sterol 14α-demethylase. ^7^ The cluster grouping of the ABCG family is based on previous reports by Lamping et al. [69] and James et al. [46]. ^8^ AtrR transcription factor orthologs have been reported to mediate azole resistance in *A. fumigatus* [34,35] and *F. graminearum* [36]. ^9^ The novel zinc-cluster transcription factor, FgSR, is the master regulator of ergosterol biosynthesis in *F. graminearum* [33].

## Data Availability

The RNA-seq raw data can be accessed at the NCBI Short Read Archive with accession number SRS14719455. GenBank accession numbers for the manually curated *F. keratoplasticum* 2781 mRNA transcript sequences are provided in Supplementary File S1.

## Supplementary Materials

The following supporting information can be downloaded at: https://www.mdpi.com/article/10.3390/jof8101070/s1, Figure S1: Phylogeny (midpoint rooted) of (a) *erg7*, *cyp51*, *erg24* and *erg25/26/27* complex genes, (b) *ebp*, *erg6*, *erg2*, *erg3*, *erg5* and *erg4*, (c) *dhcr7* and *dhcr24* and of (d) mevalonate pathway genes *erg10*, *erg13* and *hmg* with duplicated genes in some or all investigated species. Supplementary File S1: Inventory for all *F. keratoplasticum* (ergo)sterol biosynthesis, ABC transporter and transcription factor gene transcripts and their orthologs in *F. solani*, *F. vanettenii*, *F. graminearum*, *A. fumigatus* and *S. cerevisiae*. Supplementary File S2: A list of significant DEGs in *F. keratoplasticum* 2781 cells grown in the presence of VRC (16 mg/L) for 80 min.
