# Supplementary material for: Voriconazole Treatment Induces a Conserved Sterol/Pleiotropic Drug Resistance Regulatory Network, including an Alternative Ergosterol Biosynthesis Pathway, in the Clinically Important FSSC Species, Fusarium keratoplasticum
Source: J Fungi (Basel). 2022 Oct 12;8(10):1070. doi: 10.3390/jof8101070 (PMC9605146; doi:10.3390/jof8101070)
Supplement: Supplementary file 1 [file jof-08-01070-s001.zip › jof-1933587-Supplementary.pdf]

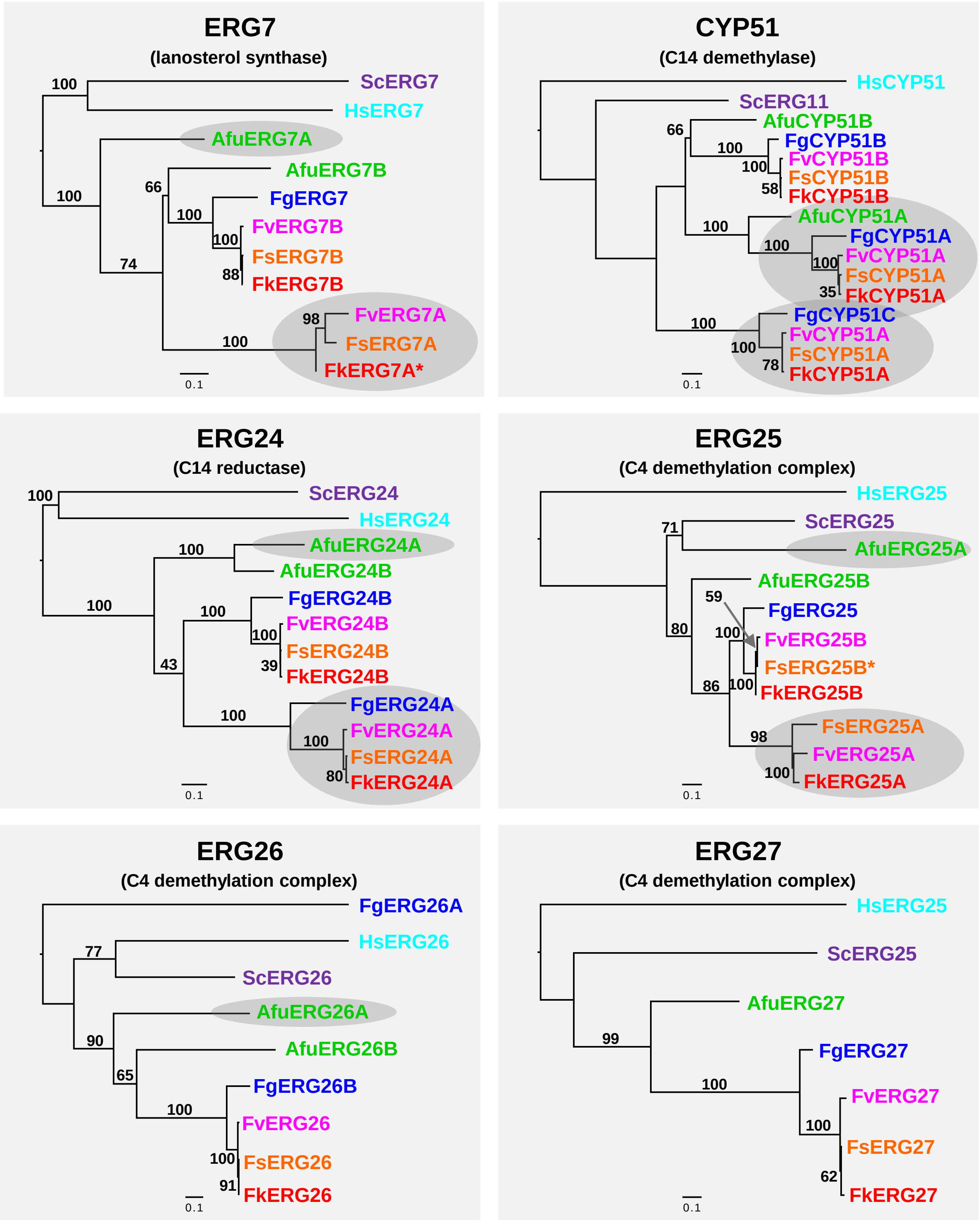

Figure S1a. Phylogeny of all *erg7*, *cyp51* (*ERG11*), *erg24* and *erg25/26/27* complex homologs (midpoint rooted). The grey-shaded areas highlight *erg* orthologs that have possibly evolved novel substrate specificities. Species name abbreviations and their designated colours are as follows: *Homo sapiens* (Hs; cyan), *Saccharomyces cerevisiae* (Sc; purple), *Aspergillus fumigatus* (Afu; green), *Fusarium graminearum* (Fg; blue), *F. vanettenii* (Fv; pink), *F. solani sensu stricto* FSSC5 (Fs; orange), *F. keratoplasticum* (Fk; red). * = partial sequence only. The percentage bootstrap support of 100 replicates is shown for all branches. The scale bars indicate the number of amino acid substitutions per position.

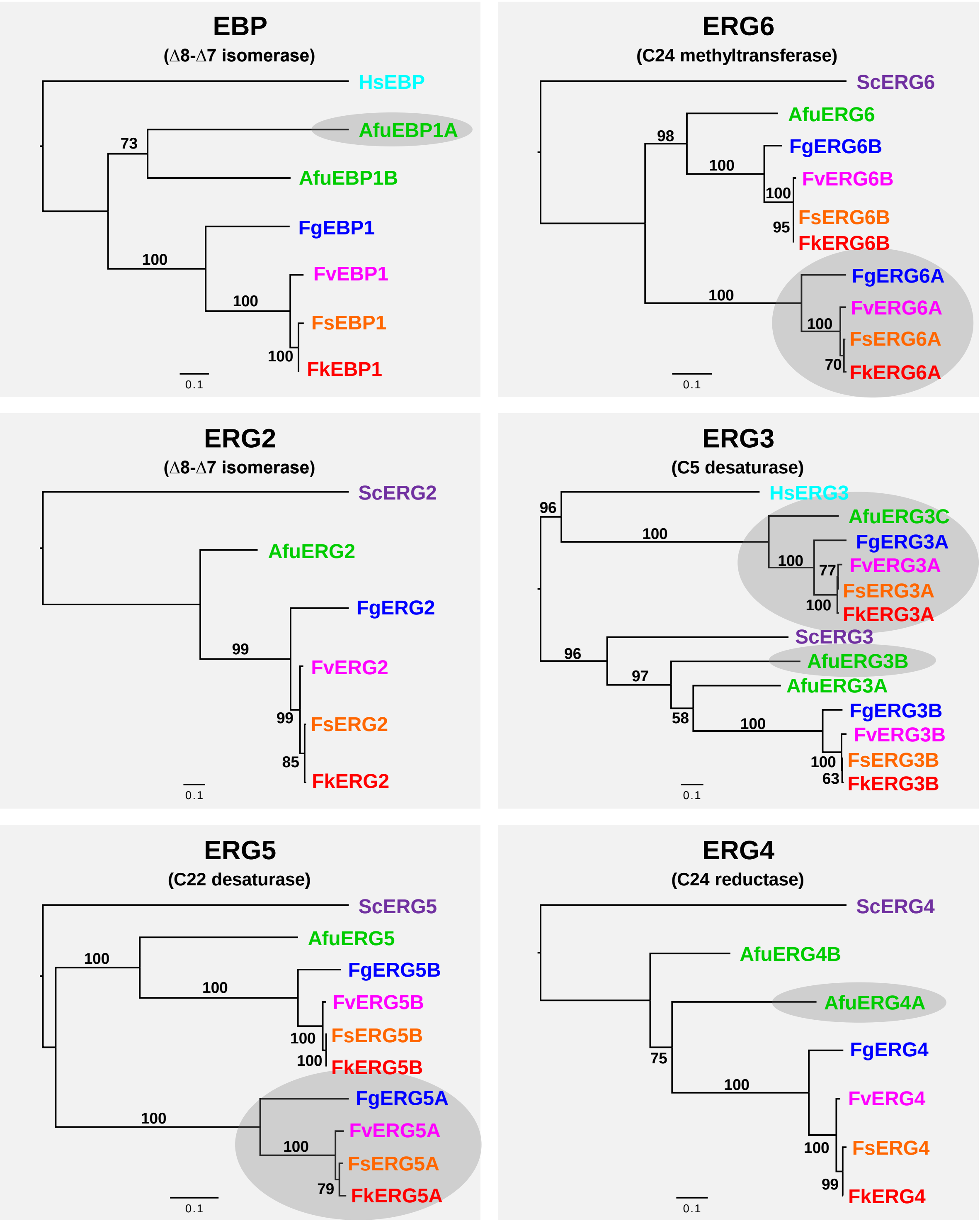

Figure S1b. Phylogeny of *ebp*, *erg6*, *erg2*, *erg3*, *erg5* and *erg4* homologs (midpoint rooted). The grey-shaded areas highlight *erg* orthologs that have possibly evolved novel substrate specificities. Species name abbreviations and their designated colours are as follows: *Homo sapiens* (Hs; cyan), *Saccharomyces cerevisiae* (Sc; purple), *Aspergillus fumigatus* (Afu; green), *Fusarium graminearum* (Fg; blue), *F. vanettenii* (Fv; pink), *F. solani sensu stricto* FSSC5 (Fs; orange), *F. keratoplasticum* (Fk; red). The percentage bootstrap support of 100 replicates is shown for all branches. The scale bars indicate the number of amino acid substitutions per position.

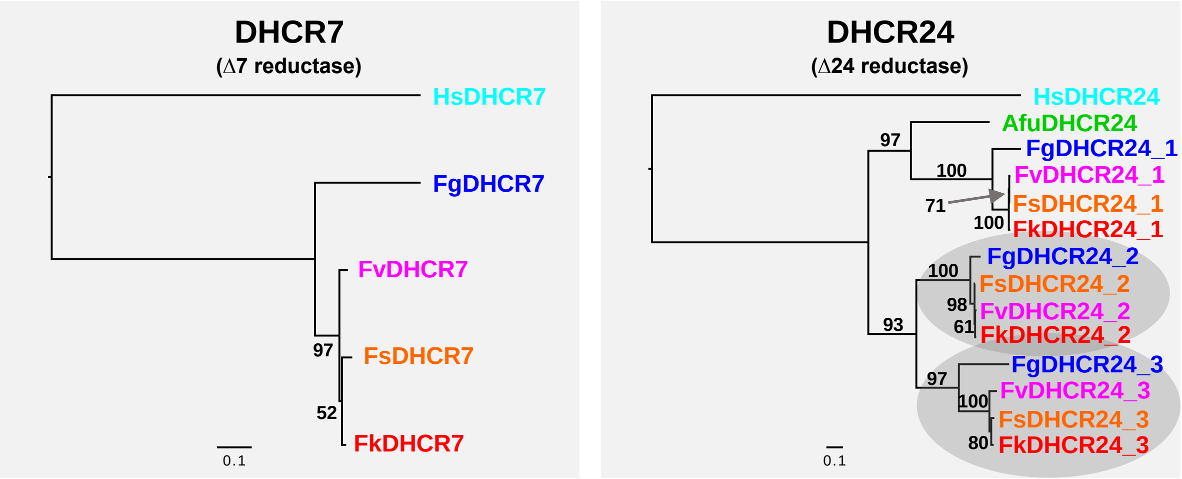

Figure S1c. Phylogeny of all human *dhcr7* and *dhcr24* homologs (midpoint rooted). The grey-shaded areas highlight *erg* orthologs that have possibly evolved novel substrate specificities. Species name abbreviations and their designated colours are as follows: *Homo sapiens* (Hs; cyan), *Saccharomyces cerevisiae* (Sc; purple), *Aspergillus fumigatus* (Afu; green), *Fusarium graminearum* (Fg; blue), *F. vanettenii* (Fv; pink), *F. solani sensu stricto* FSSC5 (Fs; orange), *F. keratoplasticum* (Fk; red). The percentage bootstrap support of 100 replicates is shown for all branches. The scale bars indicate the number of amino acid substitutions per position.

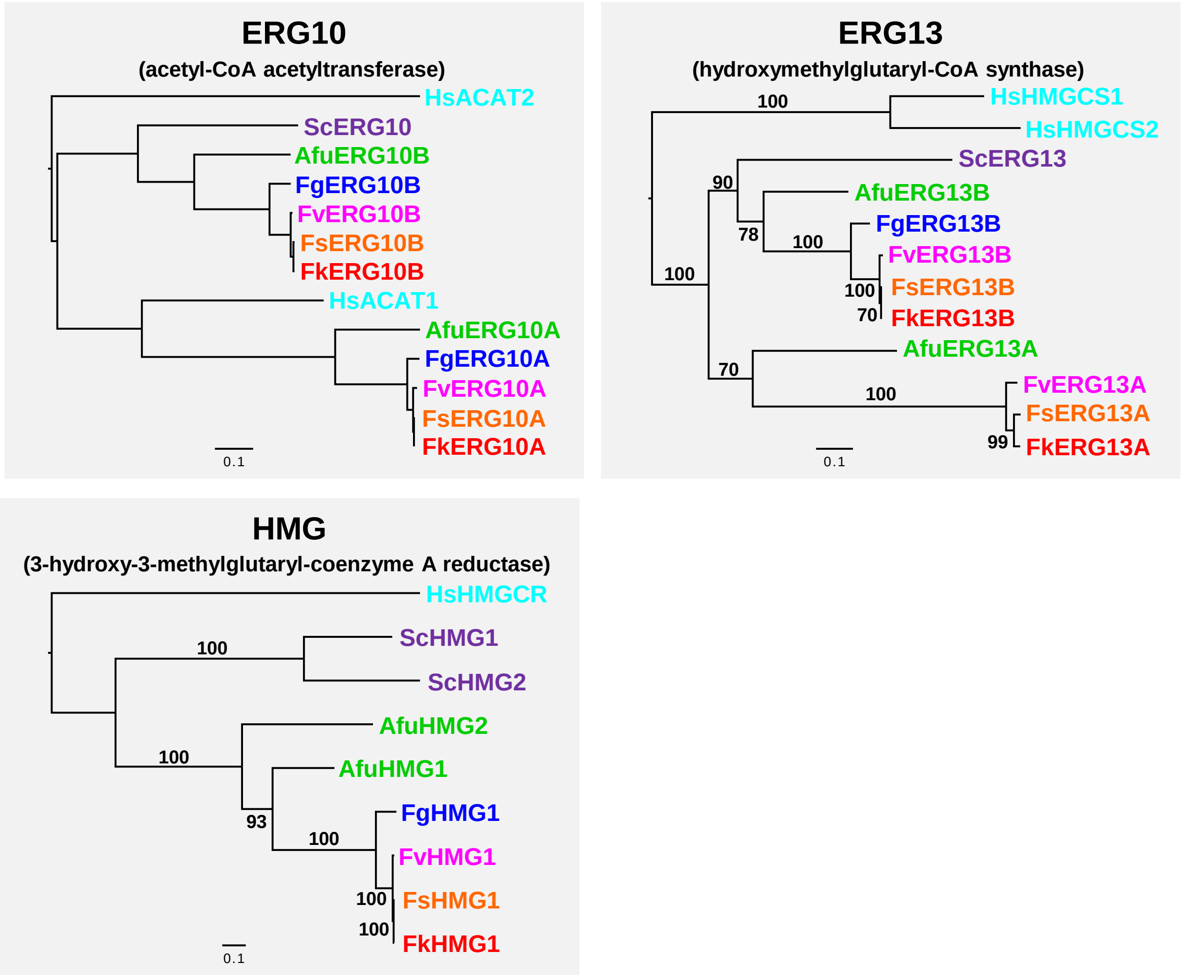

Figure S1d. Phylogeny of all *erg10*, *erg13*, and *hmg* homologs (midpoint rooted). All other mevalonate pathway genes are not duplicated and therefore excluded from the phylogenetic comparisons. Species name abbreviations and their designated colours are as follows: *Homo sapiens* (Hs; cyan), *Saccharomyces cerevisiae* (Sc; purple), *Aspergillus fumigatus* (Afu; green), *Fusarium graminearum* (Fg; blue), *F. vanettenii* (Fv; pink), *F. solani sensu stricto* FSSC5 (Fs; orange), *F. keratoplasticum* (Fk; red). The percentage bootstrap support of 100 replicates is shown for all branches. The scale bars indicate the number of amino acid substitutions per position.
